# Construction of Ternary Ce Metal–Organic Framework/Bi/BiOCl Heterojunction towards Optimized Photocatalytic Performance

**DOI:** 10.3390/nano14161352

**Published:** 2024-08-15

**Authors:** Teng Gao, Hongqi Chu, Shijie Wang, Zhenzi Li, Wei Zhou

**Affiliations:** Shandong Provincial Key Laboratory of Molecular Engineering, School of Chemistry and Chemical Engineering, Qilu University of Technology (Shandong Academy of Sciences), Jinan 250353, China; gt220539@163.com (T.G.); hqchu@qlu.edu.cn (H.C.); wsj0924@qlu.edu.cn (S.W.)

**Keywords:** photocatalysis, ternary heterojunction, BiOCl, Ce-MOF

## Abstract

Photocatalysis is the most promising green approach to solve antibiotic pollution in water, but the actual treatment effect is limited by photocatalytic activity. Herein, Bi and BiOCl were loaded onto the surface of Ce-MOF (metal–organic framework) using an electrostatic adsorption method, and a special ternary heterojunction of Ce/Bi/BiOCl was successfully prepared as a photocatalyst for the degradation of tetracycline (TC). FTIR demonstrated that the obtained photocatalyst contains functional groups such as -COOH belonging to Ce-MOF and characteristic crystal planes of Bi and BiOCl, indicating the successful construction of a ternary photocatalyst. The results of UV–vis absorption spectra confirm that the band gap of Ce/Bi/BiOCl heterojunction is reduced from 3.35 eV to 2.7 eV, resulting in an enhanced light absorption capability in the visible light region. The special ternary heterojunction constructed by Ce-MOF, Bi, and BiOCl could achieve a narrow band gap and reasonable band structure, thereby enhancing the separation of photogenerated charges. Consequently, the photocatalytic performance of the Ce/Bi/BiOCl ternary heterojunction was significantly enhanced compared to Ce-MOF, Bi, and BiOCl. Therefore, Ce/Bi/BiOCl can achieve a photocatalytic degradation rate of 97.7% within 20 min, which is much better than Bi (14.8%) and BiOCl (67.9%). This work successfully constructed MOF-based ternary photocatalysts and revealed the relationship between ternary heterojunctions and photocatalytic activity. This provides inspiration for constructing other heterogeneous catalysts for use in the field of photocatalysis.

## 1. Introduction

The development of global industry has led to increased environmental pollution [1,2]. Various organic pollutants produced by the printing and dyeing industry, the pharmaceutical industry, and the textile industry have been detected in water [3]. In addition, the widespread use of antibiotics has aroused widespread concern. This is because antibiotics used in the animal husbandry, agricultural, medical, and other industries cannot be completely metabolized by humans and animals [4,5]. These residual antibiotics enter the environment through excrement, but most wastewater treatment plants, including drinking water treatment plants, do not remove high-polarity antibiotics. Even trace amounts of antibiotics can induce bacterial resistance, and the accumulation of antibiotics in the environment can cause harm to the ecosystem, which is a serious threat to human health [6]. Tetracycline hydrochloride (TC) is one of the most commonly used antibiotics to promote animal growth and prevent disease. In the past few decades, strategies such as biological treatment, adsorption, membrane filtration, and photocatalytic degradation have been adopted to remove TC from the environment [7,8,9]. The adsorption strategy is simple but has the problems of a single performance and low regeneration rate, which limits its application in environmental treatment [10]. Therefore, the combination of adsorption and catalysis can effectively eliminate the residual TC in water [11]. Photocatalytic degradation technology has the advantages of high universality, being green and pollution-free, low energy consumption, renewable energy, a simple process, and high treatment efficiency, and it has broad application prospects in water pollution control [12].

Metal–organic framework materials (MOFs), porous materials synthesized by the coordination of metal nodes and organic ligands, have been widely studied in the field of photocatalysis due to their structural advantages, such as a large specific surface area, adjustable pore size, high porosity, and abundant active sites [13,14,15]. Xia et al. investigated and summarized the research progress on the photocatalytic degradation of organic pollutants by MOF-based materials and expounded the practical application, limitations, synthesis methods, and future development trends in MOFs [15]. The metal cerium (Ce) is mainly expressed as trivalent and quadrivalent Ce^3+^/Ce^4+^ cycles under certain specific conditions, which can provide more opportunities for electron transfer [16], and Ce has a strong oxidation capacity, high stability, and unique luminescence characteristics. Therefore, MOFs with a Ce atom as the catalytic active center have been widely used in photocatalysis and electrocatalysis, and they are more conducive to the degradation of pollutants [17,18]. However, most MOFs have the disadvantages of low conductivity and high electron–hole recombination rate, which limits their applications in photocatalysis [19,20]. Therefore, various strategies have been adopted to enhance the photocatalytic potential of MOFs, such as forming heterostructures with semiconductor materials and regulating organic ligands [21,22]. Liu et al. summarized the achievements made in the construction of heterojunction photocatalysts based on semiconductor materials and expounded its basic principles, synthesis, applications, and challenges [23]. For example, Goudarzi et al. incorporated Ce-MOF (g-C_3_N_4_/Ce-MOF) on g-CN to construct S-type heterojunction, accelerate charge transfer efficiency, and effectively separate photogenerated electron–hole pairs. In addition, the combination of g-CN and Ce-MOF increases the specific surface area of the catalyst, reduces the band gap, increases the absorption range of visible light, and makes more effective use of visible light, thus enhancing the photocatalytic Cr (VI) reduction activity [24].

In addition, semiconductor photocatalysts show great potential for TC degradation due to low cost, high efficiency, suitable band structure, and high quantum efficiency. Bi-based photocatalysts have an excellent visible light response, such as the BiOI thin-film catalyst studied by Laura et al., which efficiently removes water-based antibiotics under the activation of peroxonosulfate (PMS) and reveals the synergistic effect between PMS and BiOi [25]. In addition, Bismuth oxychloride (BiOCl), as a semiconductor photocatalytic material, has a tetragonal staircase structure, which exhibits high stability, high oxidation capacity, no pollution, and fast photogenerated carrier separation and transfer efficiency [26,27], and can directly degrade TC by sunlight [28,29]. However, BiOCl has a wide band gap (3.4 eV) and low sunlight utilization rate, which limits its actual degradation efficiency [30,31]. Therefore, some methods have been studied to compensate for the defects of BiOCl, among which metal doping and the construction of heterogeneous structures are considered to be promising methods [32,33,34]. Metal doping can form a Schottky barrier between semiconductors and inhibit the recombination of electrons and holes, which can improve the optical absorption rate and enhance the photocatalytic performance of BiOCl. Metallic Bi has the characteristics of low cost, high carrier activity, surface plasmon resonance (SPR), etc., and Bi is homologous to BiOCl and the lattice will be highly matched [35,36]. The results show that Bi modification can improve the degradation efficiency of BiOCl [37,38]. In Ding et al., a Bi/BiOI/BiOCl Z-type photocatalyst was constructed to enhance the light absorption range of the catalyst, which improved the light absorption capacity of BiOCl and promoted the photocatalytic degradation efficiency [38].

In view of this, we combined Ce-MOF, Bi, and BiOCl together to construct a ternary heterojunction for use as a heterojunction photocatalyst for catalytic TC. The introduction of Ce-MOF and Bi/BiOCl endows Ce/Bi/BiOCl with a relatively large surface area and appropriate band structure, which provided a fast charge transfer channel in the photocatalytic process to suppresses electron reflux and accelerated the separation of electrons and holes. Subsequently, we evaluated the catalytic performance of the composite photocatalyst using various characterization techniques, providing some reference support for the further exploration and synthesis of efficient catalysts in the field of photocatalytic degradation.

## 2. Materials and Methods

### 2.1. Chemicals

1,4-terephthalic acid (H_2_BDC), tetracycline (TC), (NH_4_)_2_Ce(NO_3_)_6_, Bi(NO_3_)_3_·5H_2_O, and KCl were purchased from Aladdin. Acetone, ethanol, Nafion solution, and N,N-dimethylformamide (DMF) were purchased from Sigma–Aldrich(Shanghai, China). All materials used had not been purified and reached analytical grade (99%).

### 2.2. Methods

#### 2.2.1. Synthesis of Ce-MOF

First, 116.9 mg of (NH_4_)_2_Ce (NO_3_)_6_ (213 µ mol) was dissolved in 400 µ L of water. Then, 35.4 mg of H_2_BDC (213 µ mol) was dissolved in 1.2 mL of DMF. The above two solutions were mixed and then transferred to a glass reaction tube (volume 15 mL). The glass reactor tube was heated at 100 °C via stirring using an aluminum heating block for 15 min.

After centrifugation and washing with DMF and acetone several times, the resulting precipitant was dried in air at 70 °C overnight to finally obtain Ce-MOF.

#### 2.2.2. Synthesis of Bi/BiOCl

First, 2.9 g of Bi (NO_3_)_3_·5H_2_O and 0.44 g of KCl were added to 32 mL of DMF solvent, followed by stirring for 0.5 h. After the reaction was complete, the samples were washed with water and ethanol, respectively, and finally dried at 70 °C for 12 h. The collected samples were represented as Bi/BiOCl. Then, the aqueous solution was transferred into a 50 mL volume Teflon-lined stainless autoclave and maintained continuously at 160 °C for 12 h. After being washed with deionized water and ethanol, respectively, and dried at 70 °C under vacuum, the final Bi/BiOCl product was obtained.

#### 2.2.3. Synthesis of Ce-MOF/Bi/BiOCl

Ce-MOF was mixed with Bi/BiOCl in DMF solution at a mass ratio of 2.5:1 and sonicated for 1 h to obtain Ce-MOF/Bi/BiOCl, abbreviated as Ce/Bi/BiOCl.

### 2.3. Characterizations

The prepared Ce-MOF, Bi/BiOCl, BiOCl, and Ce/Bi/BiOCl catalysts were examined to reveal crystalline phase structures by X-ray diffraction (XRD) (Rigaku, Tokyo, Japan). Transmission electron microscopy (TEM) (FEI Talos F200X, Washington, DC, USA) and field emission scanning electron microscopy (SEM) (HITACHI, SU8010, Tokyo, Japan) were used to reveal the microstructures of the Ce-MOF, Bi/BiOCl, BiOCl, and Ce/Bi/BiOCl samples. The N_2_ adsorption isotherm of the Ce/Bi/BiOCl sample was obtained to analyze the pore size distribution and the specific surface area (FEI ESCALAB250, Washington, DC, USA) by the Brunauer–Emmett–Teller (BET) method. The photoluminescence (PL) spectra (Edinburgh, livingston, FLS 980, Scotland, UK) were determined to analyze the carrier recombination (excitation wavelength 375 nm). The UV-Vis diffuse reflectance spectroscopy was measured on SolidSpec-3700 (DRS, Shimadzu, Tokyo, Japan).

### 2.4. Measurement of Photocatalytic Activity

The photocatalytic performance of Ce/Bi/BiOCl, BiOCl, and Bi/BiOCl was tested by photocatalytic degradation tetracycline (TC) under simulated visible light (AM 1.5 cut-off filter). As usual, 50 mg of prepared catalyst was added to 100 mL of TC solution (40 mg/L). A 300 W Xenon arc lamp was used to provide the simulated visible light. Before irradiation, the catalyst was reacted in a dark environment for 10 min to reach adsorption/desorption equilibrium. During the photocatalytic reaction, 4 mL was extracted from the solution every 5 min and the concentration of TC was measured via a UV–visible spectrophotometer at 357 nm.

## 3. Results and Discussion

### 3.1. The Crystalline Phase Structure Analysis

**Figure 1:**
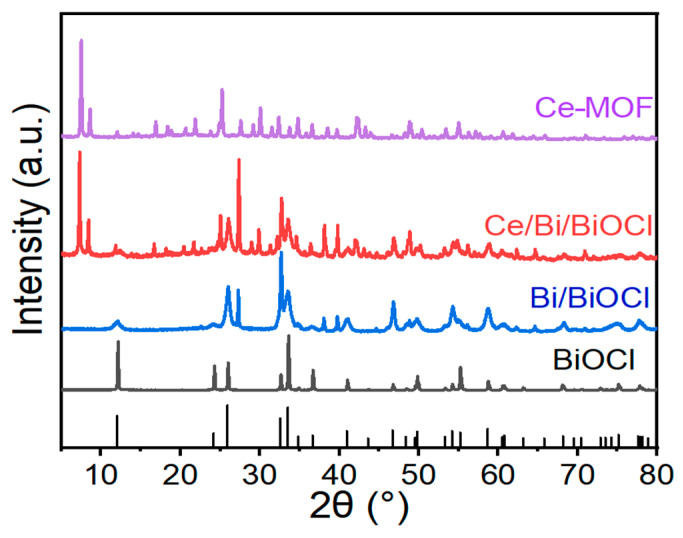
XRD patterns of Ce-MOF, Bi/BiOCl, BiOCl, and Ce/Bi/BiOCl, respectively.

To investigate the crystalline phase structures of materials, X-ray diffraction (XRD) tests were conducted. Figure 1 shows the XRD patterns of Ce-MOF, Bi/BiOCl, BiOCl, and Ce/Bi/BiOCl. From the XRD patterns of Bi/BiOCl and BiOCl, it can be seen that there are several typical peaks attributed to Bi and BiOCl, located at 2θ = 12.0, 27.2, 39.6, 46.8, 58.8, and 62.2° (JCPDS no. 73-2060 and 85-1329). The resultant solid shows the characteristic XRD pattern of the Ce-MOF material as a pure crystalline phase, which has similarities to the results of XRD in the literature [39]. The XRD pattern of synthesized Ce/Bi/BiOCl is highly crystalline, which is consistent with the results of Ce-MOF, Bi/BiOCl, and BiOCl. Thus, the XRD analyses confirmed that Ce/Bi/BiOCl is composed of Ce-MOF and Bi/BiOCl.

### 3.2. The Morphology Analysis

**Figure 2:**
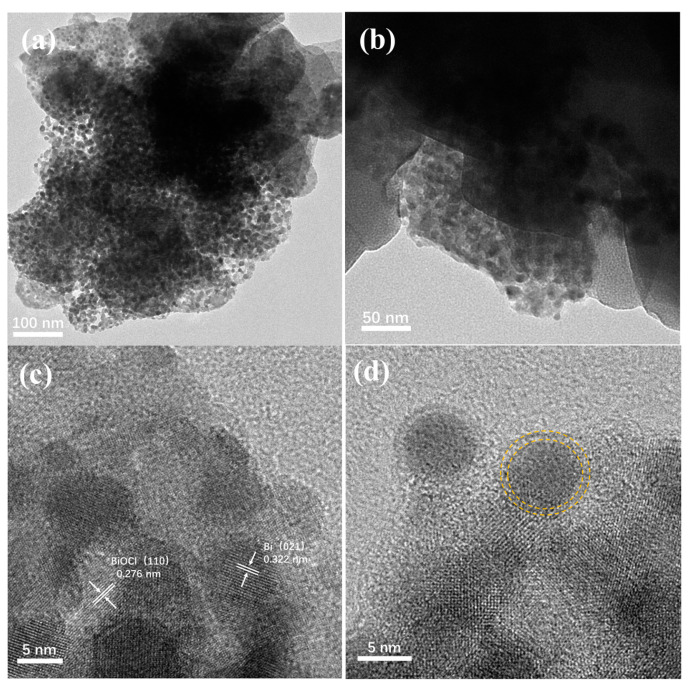
TEM images (**a**,**b**) and high-resolution TEM images (**c**,**d**) of Ce/Bi/BiOCl.

The morphology of synthesized Ce/Bi/BiOCl was characterized by TEM. As shown in Figure 2a, it can be seen that the structure of the block for Ce/Bi/BiOCl is obtained, which is composed of blocks and a large number of small particles. In addition, the interface between particles and blocks can be observed (Figure 2b), which is generated after the recombination of Ce-MOF, Bi, and BiOCl.

As shown in Figure 2c, the 0.276 nm lattice fringe matches the (110) crystal plane of BiOCl. Meanwhile, the lattice stripe of 0.322 nm belongs to the (021) crystal plane of Bi, indicating that the main component of the small particles is Bi. The particle size obtained from TEM results is approximately 5 nm (Figure 2d). Moreover, there is a clear shell on the outer layer of the particles that provides protection to prevent photo etching. Through the morphological analysis of Ce/Bi/BiOCl, the Bi/BiOCl is successfully compounded on the surface of Ce-MOF. The combination of Ce/Bi/BiOCl can not only increase the generation of photogenerated charge carriers but also greatly increase the adsorption active sites, giving it stronger photocatalytic degradation performance.

### 3.3. The N_2_ Adsorption Analysis

**Figure 3:**
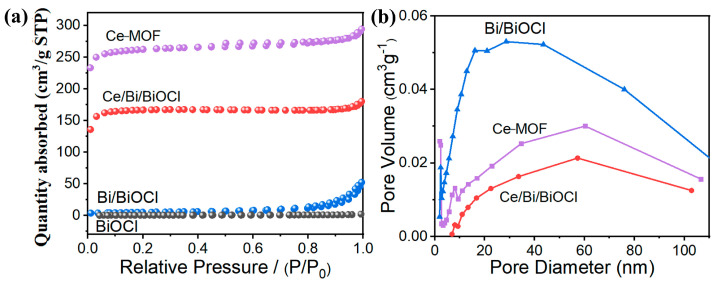
(**a**) N_2_ adsorption/desorption isotherms of Ce-MOF, Bi/BiOCl, BiOCl, and Ce/Bi/BiOCl. (**b**) Pore size distribution curves of Ce-MOF, Bi/BiOCl, and Ce/Bi/BiOCl.

After combining Ce-MOF and Bi/BiOCl, the changes in specific surface area and pore size were studied through N_2_ adsorption/desorption experiments. As expected, Ce-MOF exhibits the highest specific surface area, which is advantageous for the adsorption of TC (Figure 3a). Compared to BiOCl, the presence of Bi did not increase the specific surface area of Bi/BiOCl. Therefore, compared to Ce-MOF, Bi and BiOCl do not have an advantage in specific surface area. Moreover, Ce/Bi/BiOCl maintains a high specific surface area even after compounding Ce-MOF and Bi/BiOCl.

According to the pore size distribution curves (Figure 3b), the pore size of the original Ce-MOF is mainly distributed in the mesoporous region, around 7.5 nm. However, the pore size of Bi/BiOCl is mainly distributed in the large pore area (Table 1). Therefore, the pore size distribution of the composite material Ce/Bi/BiOCl is in two regions, mesopores and macropores, which enables pollutant molecules to contact the catalytic active sites through the pores and efficiently utilize photogenerated charge carriers [40].

### 3.4. The XPS and FT-IR Analysis

**Figure 4:**
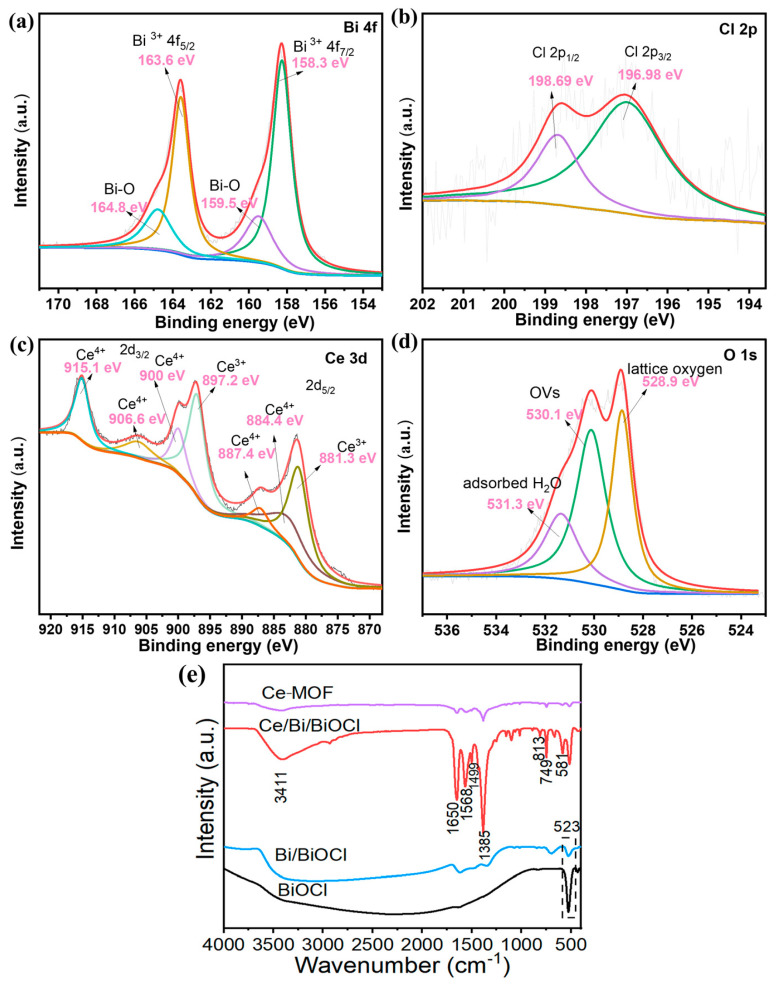
XPS spectra of Ce/Bi/BiOCl: (**a**) Bi 4f, (**b**) Cl 2p, (**c**) Ce 3d, and (**d**) O 1 s. The XPS characterization was performed on Ce/Bi/BiOCl to probe the chemical states. (**e**) FTIR spectra of Ce-MOF, Bi/BiOCl, BiOCl, and Ce/Bi/BiOCl, respectively.

In Figure 4a, two prominent peaks at 163.6 and 158.3 eV correspond to Bi 4f 7/2 and Bi 4f 5/2, proving that Bi exists as Bi^3+^ in Ce/Bi/BiOCl. In addition, the peaks situated at positions of 159.5 and 164.8 eV are attributed to the B-O bond in BiOCl. The two prominent peaks at 196.98 and 198.69 eV correspond to Cl 2p 3/2 and Cl 2p 3/2 (Figure 4b). The peaks located at 881.3, 884.4, 887.4 eV, 897.2, 900, 906.6, and 915.1 eV can be attributed to Ce 3d5/2 and Ce 3d3/2, reflecting that Ce exists as Ce^3+^ and Ce^4+^ in Ce/Bi/BiOCl (Figure 4c). The O1s spectrum of Ce/Bi/BiOCl can be fitted to three peaks at 528.9, 530.1, and 531.3 eV, corresponding to lattice oxygen, OVs, and adsorbed H_2_O, respectively (Figure 4d).

The FT-IR spectra of Ce-MOF, Bi/BiOCl, BiOCl, and Ce/Bi/BiOCl are shown in Figure 4e. The spectrum of Ce-MOF displays that the adsorption bands at 1650 cm^−1^ and 1385 cm^−1^ belong to the asymmetric carboxylate group (νas (COO^−^)) and the aromatic ring (ν (C=C)_ar_), which can be observed in Ce/Bi/BiOCl, proving the existence of Ce-MOF after recombination [41]. The peak at 523 cm^−1^ is owed to the deformation vibration of Bi-O. The peak at 1621 cm^−1^ is attributed to tensile and bending vibrations of -OH caused by the surface hydration of the sample. In addition, the spectrum of Ce/Bi/BiOCl also exhibited characteristic peaks of Bi/BiOCl at 1499, 813, and 749 cm^−1^. The above results provide strong evidence of the efficient complexation of Ce/Bi/BiOCl.

### 3.5. The UV-Vis Analysis

**Figure 5:**
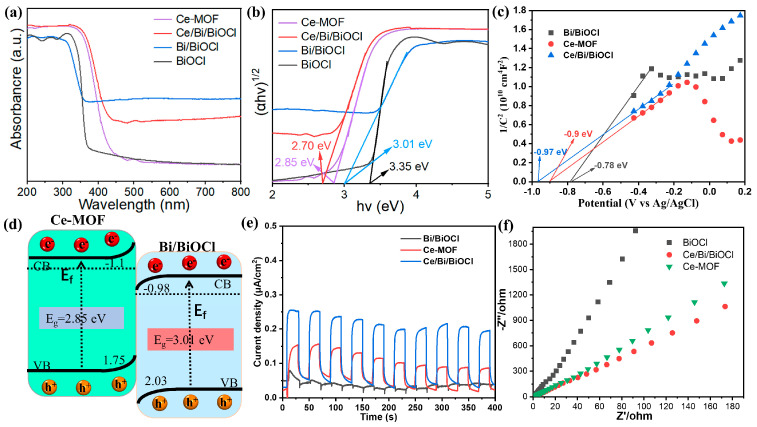
(**a**) Ultraviolet–visible absorption spectra of Ce-MOF, Bi/BiOCl, BiOCl, and Ce/Bi/BiOCl and (**b**) the corresponding Kubelka–Munk conversion reflection spectra. (**c**) Mott–Schottky test. (**d**) Energy diagram. (**e**) Transient photocurrent responses. (**f**) EIS Nyquist plots.

In order to gain a deep understanding of the effect of Bi/BiOCl and Ce/Bi/BiOCl coupling on photocatalytic activity, UV–visible absorption spectroscopy was conducted. The UV–vis absorption spectra of Ce-MOF, BiOCl, and Bi/BiOCl shown in Figure 5a display strong absorption in the UV spectrum region (200−400 nm) while the absorption is weakened in the visible light spectrum region (400–800 nm). In comparison, Ce/Bi/BiOCl not only exhibits significant absorption in the ultraviolet region but also shows a noticeable redshift phenomenon, where the absorption edge shifts towards the infrared region. These phenomena indicate that the coupling effect between Bi/BiOCl and Ce-MOF expands the absorption range of Ce/Bi/BiOCl, which is beneficial for improving photocatalytic performance.

In addition, the band gaps of Ce-MOF, Bi/BiOCl, BiOCl, and Ce/Bi/BiOCl were calculated by the Kubelka–Munk method (α Hv = A (hv − Eg)^2^, where α is the absorption coefficient, Eg is the band gap energy, v is the optical frequency, h is the Planck constant, and A is a constant) [42]. The plots of (αhν)^2^ versus (hν) derived from the UV–vis absorption spectra are shown in Figure 5b. The calculated band gaps of Ce-MOF, Bi/BiOCl, BiOCl, and Ce/Bi/BiOCl were determined to be ~2.85 eV, 3.01 eV, 3.35 eV, and 2.7 eV, respectively. The above results exhibit that Ce/Bi/BiOCl possess the smallest band gap compared to Ce-MOF, Bi/BiOCl, and BiOCl, illustrating that the synergistic effect between Ce-MOF and Bi/BiOCl optimizes the band structure of the composite material, which is beneficial for the utilization of visible light. The flat band potentials of Ce-MOF, Bi/BiOCl, and Ce/Bi/BiOCl are obtained from the Mott–Schottky plots shown in Figure 5c, which are −0.9, −0.97, and −0.78 eV, respectively. Therefore, the conduction band potentials (ECB) of Ce-MOF, Bi/BiOCl, and Ce/Bi/BiOCl are −1.1, −1.17, and −0.98 eV, which are 0.2 eV lower than the flat band potentials. Thus, the energy bands of the Ce-MOF and Bi/BiOCl exhibit the characteristic charge transfer path of the Z-scheme heterojunction, which facilitates the separation and transfer of photogenerated carriers (Figure 5d). The transient photocurrent response and EIS Nyquist plot demonstrate that the Ce/Bi/BiOCl has the maximum photocurrent value and optimal charge transfer dynamics, which is consistent with the conclusion obtained from the energy-level structure of the material (Figure 5e,f).

### 3.6. The Photocatalytic Performance

**Figure 6:**
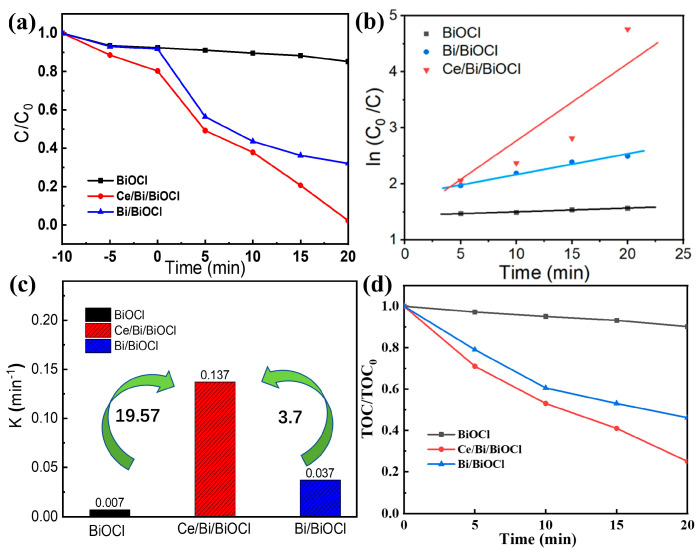
(**a**) Photocatalytic activities, (**b**) kinetic analysis, and (**c**) pseudo-first-order kinetic constants of Bi/BiOCl, BiOCl, and Ce/Bi/BiOCl, respectively. (**d**) Mineralization of TC over Bi/BiOCl, BiOCl, and Ce/Bi/BiOCl.

The photocatalytic activities of Bi/BiOCl, BiOCl, and Ce/Bi/BiOCl heterojunction were evaluated using TC as the photocatalytic degradation target under simulated visible light irradiation. As shown in Figure 6a, to evaluate the adsorption/desorption of TC pollutants, the Bi/BiOCl, BiOCl, and Ce/Bi/BiOCl were subjected to dark treatment to achieve adsorption/desorption equilibrium.

According to the results, the physical adsorption rate of the Ce/Bi/BiOCl composite material in a dark environment is around 20%, which may be attributed to the optimal combination of material pore size, adsorption, and surface area and interaction sites derived from Ce-MOF, Bi, and BiOCl. Subsequently, the Bi/BiOCl, BiOCl, and Ce/Bi/BiOCl were subjected to the degradation of TC under simulated sunlight. As shown by the results, the heterojunction constructed after the composite of Ce-MOF and Bi/BiOCl significantly enhances the photocatalytic activity, with a degradation rate increase from 14.8% and 67.9% to 97.7% within 20 min, respectively. Moreover, the catalytic performance of Ce/Bi/BiOCl is superior to most other similar catalysts (Table 2). The kinetic reaction process of TC photocatalytic degradation for Bi/BiOCl, BiOCl, and Ce/Bi/BiOCl was simulated using the following formula: ln (C/C_0_) = −kt. From the simulation results (Figure 6b), the curves of Bi/BiOCl, BiOCl, and Ce/Bi/BiOCl obtained from ln (C/C_0_) and irradiation time (t) follow a first-order linear pattern, and the corresponding value of the rate constant k is shown in Figure 6c. Significantly, Ce/Bi/BiOCl possesses the highest rate constant k (0.137 min^−1^), which is about 19.57 and 3.7 times higher than Bi (0.007 min^−1^) and BiOCl (0.0037 min^−1^), respectively. The synergistic effect of Ce/Bi/BiOCl ternary complexes in the photocatalytic process is quantified by the synergistic index: Ce/Bi/BiOCl is greater than Bi/BiOCl and Bi. The above experimental results confirm that the excellent photocatalytic activity of Ce/Bi/BiOCl may be due to its large specific surface area and reasonable band structure, such as a narrow band gap. More importantly, the rational construction of the Ce/Bi/BiOCl ternary system may enhance the separation of photogenerated charges, thereby suppressing the recombination process of electron–hole pairs. The mineralization of TC by Bi/BiOCl, BiOCl, and Ce/Bi/BiOCl was evaluated by measuring TOC variation. Within 20 min, it can be seen that Ce/Bi/BiOCl has the best mineralization rate, reaching 23.7%, which is much better than Bi/BiOCl and BiOCl (Figure 6d). This indicates that Ce/Bi/BiOCl can efficiently degrade TC in solution into CO_2_, water, and some inorganic species.

### 3.7. The Photocatalytic Mechanism and Stability Test

In order to investigate the positive role of the synergistic effect of the ternary system in Ce/Bi/BiOCl heterojunctions in-depth, an active species capture experiment in a KI/t-BuOH/AgNO_3_/BQ catalytic reaction system was carried out to reveal the active species involved in the photocatalytic reaction process, in which KI/t-BuOH/AgNO_3_/BQ were used as the trapping agents of h^+^, ·OH, e^−^, and ·O_2_^−^, respectively.

**Figure 7:**
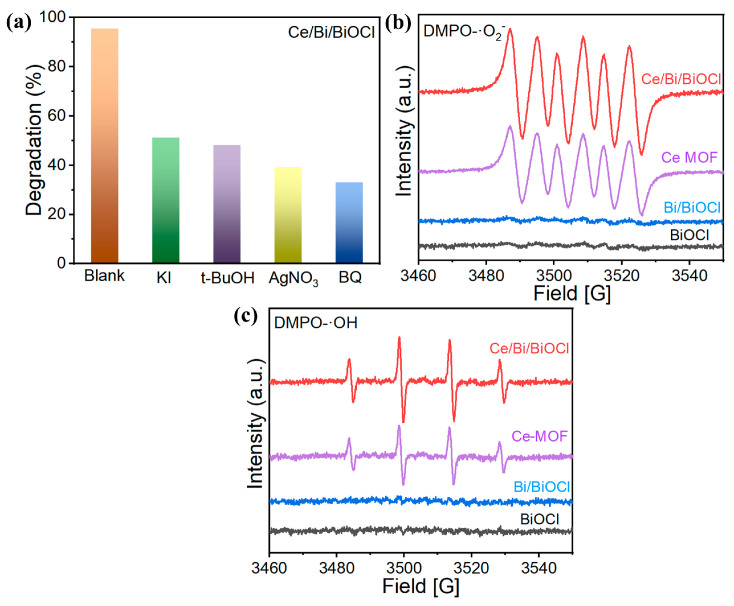
(**a**) Trapping experiments of active species for Ce/Bi/BiOCl; (**b**) EPR signals for ·O_2_^−^ and (**c**) ·OH.

The existence of KI, t-BuOH, AgNO_3_, and BQ all have a negative impact on the degradation of TC. As shown in Figure 7a, KI/t-BuOH/AgNO_3_/BQ, respectively, inhibited the removal rate of TC to 51.3%, 48.5%, 38.6%, and 32.2%. Furthermore, ESR was adopted to further investigate the photocatalytic mechanism. As depicted in Figure 7b, when Ce/Bi/BiOCl and Ce-MOF were used as photocatalysts, ESR-characteristic signals of DMPO-O^2−^ and DMPO-OH were detected under visible light irradiation, which indicated the existence of ·OH and ·O^2−^. The presence of more ·O^2−^ in the Ce/Bi/BiOCl indicates that the CBM of the Ce/Bi/BiOCl is more negative than that of Ce-MOF, Bi, and BiOCl [52,53]. Moreover, the presence of more ·OH in the Ce/Bi/BiOCl indicates that photogenerated holes in the valence band of Ce/Bi/BiOCl react with chemisorbed water molecules to produce ·OH radicals during the photocatalytic degradation process. The strong signal strengths of DMPO-O^2−^ and DMPO-OH proved the high yield of ·O^2−^ and ·OH, which are considered important active groups in the oxidation of organic pollutants.

**Figure 8:**
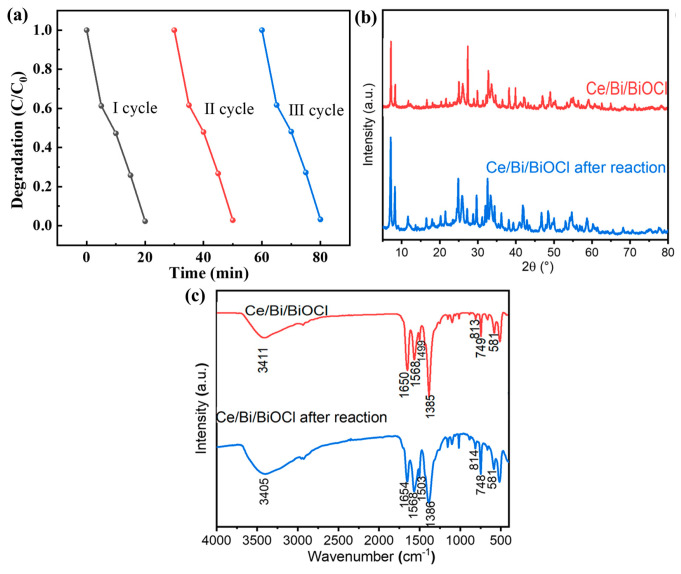
(**a**) Repeatability experiment of Ce/Bi/BiOCl photocatalytic degradation of TC. XRD (**b**) and FTIR (**c**) of Ce/Bi/BiOCl after reusability test.

As shown in Figure 8a, the repeatability experiment results indicate that the performance of Ce/Bi/BiOCl has hardly changed significantly after three consecutive photocatalytic tests, proving that Ce/Bi/BiOCl has excellent photocatalytic stability. In order to further investigate the structural stability of the catalyst, XRD and FTIR characterization were performed on Ce/Bi/BiOCl after photocatalysis. Comparing the XRD and FTIR results before and after the reusability test can prove that the structure of Ce/Bi/BiOCl has not undergone significant changes, which proves the excellent stability of Ce/Bi/BiOCl (Figure 8b,c).

## 4. Conclusions

In summary, a new Ce/Bi/BiOCl ternary heterojunction was designed and successfully prepared for photocatalytic degradation. The synergistic results of SEM, BET, and FTIR confirmed the successful construction of the Ce/Bi/BiOCl ternary heterojunction. The photocatalytic activity of the Ce/Bi/BiOCl ternary heterojunction for TC degradation was investigated. The relevant experimental results indicated that the synergistic effect of the MOF’s high specific surface area, large pore size, and multiple active sites resulted in the excellent photocatalytic degradation performance of the Ce/Bi/BiOCl. Most important of all, after combining Ce-MOF, Bi, and BiOCl, the band structure of Ce/Bi/BiOCl had been optimized; thus, the photocatalytic degradation rate increased from 14.8% and 67.9% to 97.7% within 20 min, respectively. Based on UV–visible absorption spectroscopy and EPR, it was further confirmed that the construction of Ce-MOF, Bi, and BiOCl ternary heterojunctions could achieve a narrow band gap and reasonable band structure, thereby enhancing the separation of photogenerated charges and suppressing the recombination process of electron–hole pairs. This work successfully constructed a ternary photocatalyst consisting of Ce-MOF, Bi, and BiOCl and revealed the relationship between ternary heterojunction and photocatalytic activity. Therefore, the above research has provided a novel strategy for the future preparation of MOF-based ternary heterojunctions for repairing drug antibiotics and inspires the future exploration of multi-component heterojunctions in environmental remediation.

## Figures and Tables

**Table 1:** The specific surface areas, pore sizes, and pore volumes of Ce-MOF, Bi/BiOCl, and Ce/Bi/BiOCl.

Catalysts	S_BET_ (m^2^/g)	Pore Size (nm)	Pore Volume (cm^3^/g)
S_BET_ (m^2^/g)	12.3	3.6	0.53
Pore Volume (cm^3^/g)	814	7.5	0.28
Pore Size (nm)	518	8.5	0.21

**Table 2:** Comparisons of photocatalytic activities for Ce/Bi/BiOCl and recently reported similar catalysts for the degradation of pollutants.

Photocatalysts	Pollutant	Degradation Efficiency	Kinetic Constants	Reference
Ce/Bi/BiOCl	TC	97.7% (20 min)	0.137	This work
0.2-(Zr/Ce)UiO-66 (NH_2_)@CN	TC	98% (120 min)	0.026	[43]
ZIF-67@Ce-MOF-600	TC	99.2% (30 min) (PMS)	0.073	[20]
B-TiO_2_	TC	66.2% (270 min)	4.50 × 10^−3^	[44]
BiBDC/BiVO_4_	TC	97.9% (30 min)	0.1295	[45]
BiOI/BiOBr-6	TC	90.61% (90 min)	1.0799	[28]
(BOC/WO)–Sn-Tu_1_	TC	71.68% (50 min)	0.0131	[46]
0D/2D CuO/BiOCl	TC	90.3% (80 min)	/	[47]
MOF-BiOCl/MoS	TC	90% (20 min)	0.104	[48]
Ce/FeTiO_3_	TC	97.50% (120 min)	0.02078	[49]
OD/2D TiO_2_ (B)/BiOCl	TC	86% (1 h)	0.03671	[50]
Ce/Ti-MOF	TC	90% (2 h)	0.01243	[51]

## Data Availability

The data presented in this study are available on request from the corresponding author.
